# Promises and Pitfalls of Regenerative Therapies for Androgenetic Alopecia: Platelet-Rich Plasma, Photobiomodulation, Stem Cells, and Exosomes

**DOI:** 10.3390/medsci14010005

**Published:** 2025-12-22

**Authors:** Aditya K. Gupta, Tong Wang, Ryan Welter, Robin Unger, Ricardo Mejia

**Affiliations:** 1 Ad Hoc Committee on Regenerative Medicine, International Society of Hair Restoration Surgery (ISHRS), Chicago, IL 60608, USA; r.welter@regenerismedical.com (R.W.); drrobin@ungermedical.com (R.U.); hairdocmd@aol.com (R.M.); 2 Division of Dermatology, Department of Medicine, Temerty Faculty of Medicine, University of Toronto, Toronto, ON M5S 1A1, Canada; 3 Mediprobe Research Inc., London, ON N5X 2P1, Canada; twang@mediproberesearch.com; 4 Regeneris Medical, Westwood, MA 02090, USA; 5 Department of Dermatology, Mount Sinai Medical Center, New York, NY 10029, USA; 6 Unger Medical, New York, NY 10021, USA; 7 Jupiter Dermatology and Hair Restoration, Jupiter, FL 33477, USA

**Keywords:** regenerative medicine, platelet-rich plasma, low-level light therapy, mesenchymal stem cell transplantation, exosomes, androgenetic alopecia, pattern baldness

## Abstract

Background: Regenerative therapies have emerged in recent years. In particular, their utility in managing androgenetic alopecia—the most prevalent hair loss condition worldwide, affecting up to half of adults—is an active area of research. Navigating this space can be challenging for physicians due to widespread commercialization, lack of high-quality evidence, and an evolving regulatory landscape. Objective: To critically review recently published evidence (2020–2025) on platelet-rich plasma, photobiomodulation, stem cells, and exosomes for the treatment of androgenetic alopecia. Methods: A scoping review was conducted using PubMed, Embase (Ovid) and the Cochrane Controlled Register of Trials in February and November of 2025. Combination therapies were excluded. Results and Conclusions: Platelet-rich plasma is the most studied modality, with emerging investigations into newer formulations such as leukocyte-rich and pure platelet-rich plasma. However, recent studies are limited by inconsistent reporting of cellular composition, short follow-up durations, and a lack of comparative data on treatment protocols. The efficacy of photobiomodulation as a monotherapy remains debated, with inconsistent reporting of device parameters. Stem cells and exosomes show promising, though still limited, clinical evidence in inducing hair regrowth. Standardization of these therapies is crucial, with emphasis on transparency, reproducibility, and patient safety.

## 1. Introduction

**Figure 1:**
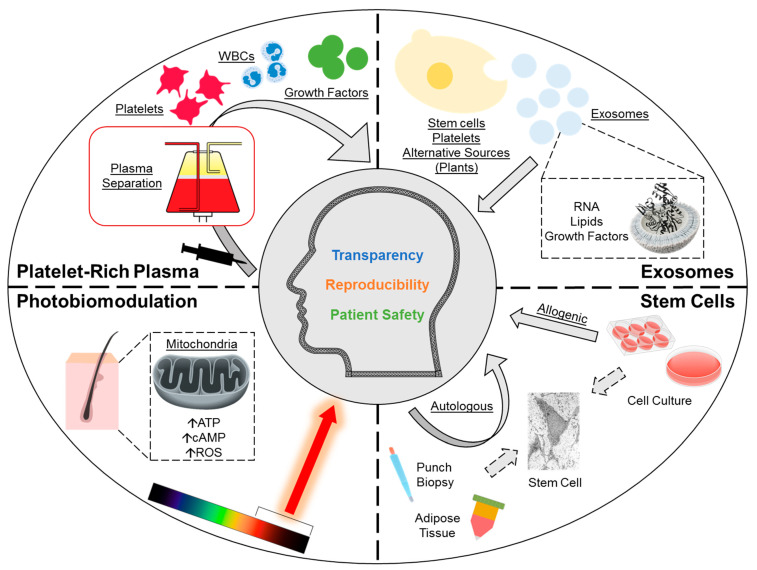
Overview of regenerative therapies for androgenetic alopecia under investigation. This includes platelet-rich plasma, photobiomodulation, stem cells and exosomes. ATP, adenosine triphosphate; cAMP, cyclic adenosine monophosphate; ROS, reactive oxygen species; WBC, white blood cells.

There is growing interest in regenerative medicine for the treatment of androgenetic alopecia (AGA) (Figure 1) [1,2,3,4,5]. AGA is a prevalent hair loss condition affecting up to half of males and up to one-fourth of females with an age-dependent increase in incidence; a growing comorbid population globally, and an emerging phenomenon of prepubescent AGA, further exacerbate the burden [6]. Current investigational therapies aiming at inducing hair follicle regeneration include platelet-rich plasma (PRP), photobiomodulation (PBM), stem cells, and extracellular vesicles (exosomes). PRP is an autologous administration of a patient’s plasma fraction—concentrated with platelets through centrifugation—and is thought to promote hair growth via the release of platelet-derived growth factors and other paracrine signaling mediators. PBM is a device-based therapy that utilizes low-power, non-thermal light sources for scalp irradiation, and is hypothesized to enhance mitochondrial function, which drives hair growth. Stem cells are being studied for both direct and indirect mechanisms of action, such as directly through the differentiation of hair follicle-derived stem cells or indirectly via paracrine modulation by adipose-derived stem cells. Lastly, exosomes—cargo-carrying vesicles found almost universally across all cell types—are recognized for their potential paracrine modulatory effects on hair growth.

PRP and PBM have already been popularized in clinical practice, especially for patients who do not respond adequately to conventional treatments. Newer investigational therapies—stem cells and exosomes—face regulatory challenges and lack robust safety data. As the clinical application of regenerative treatments has outpaced the gathering of high-quality empirical evidence, physicians have been unable to reach a consensus on how best to design and implement these therapies. In this review, we systematically gathered published clinical evidence spanning the last 5 years on the use of regenerative medicine in AGA patients. For each modality, we discuss mechanisms of action, clinical efficacy and safety profiles, as well as current challenges in clinical application.

## 2. Materials and Methods

A scoping review—restricted to publications within the last 5 years—was conducted on 27 February 2025, with a follow-up search on 6 November 2025 (Open Science Framework: https://osf.io/zjexc/). Three databases were queried: PubMed, Embase (Ovid), and the Cochrane Central Register of Controlled Trials (CENTRAL). The search strategy utilized terms and/or subject headings that included “regenerative medicine”, “guided tissue regeneration”, “platelet rich plasma”, “autologous platelet”, “platelet rich fibrin”, “autologous fibrin matrix”, “low level laser therapy”, “light therapy”, “stromal vascular fraction”, “adipose-derived stem cells”, “mesenchymal stem cell”, “multipotent stem cell”, “autologous stem cell”, “exosome”, “extracellular vesicles”, “microvesicles”, “nanovesicle”, and “cell based therapy”. Results were further limited using the search term and/or subject heading for “androgenetic alopecia” or “pattern hair loss”. Screening for additional eligible records was conducted using ClinicalTrials.gov and through reference screening of relevant review articles. The PRISMA reporting recommendations were followed [7].

Article deduplication and the screening of titles and abstracts were performed using Covidence (www.covidence.org). The inclusion criteria were studies reporting the efficacy of regenerative therapy in AGA patients, including PRP, PBM, stem cells, and exosomes. Single-arm studies evaluating combination treatment were excluded. Therapeutic products with multiple active ingredients or unclear composition were excluded. Pre-clinical studies, non-English articles, reviews, and expert opinions were also excluded.

## 3. Results and Discussion

**Figure 2:**
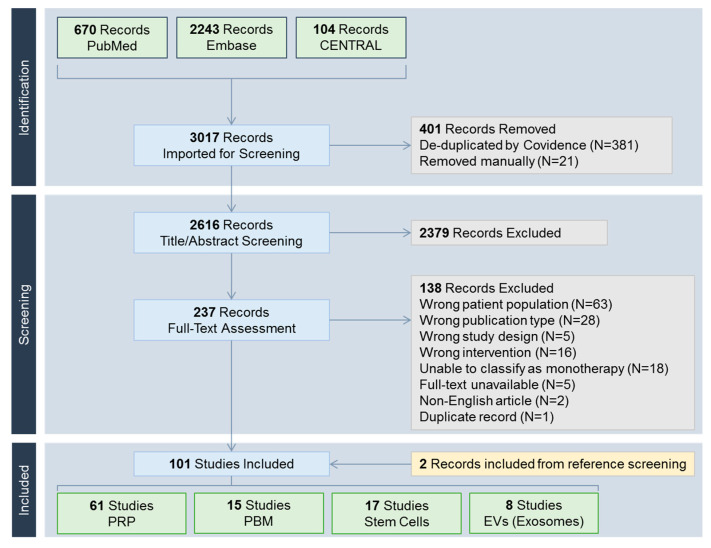
PRISMA (Preferred Reporting Items for Systematic reviews and Meta-Analyses) flow chart. CENTRAL, Cochrane Central Register of Controlled Trials; EV, extracellular vesicle; PBM, photobiomodulation; PRP, platelet-rich plasma.

A total of 3017 records were identified (Figure 2). Included studies were classified into one of the following treatment modalities: PRP including its derivatives such as platelet-rich fibrin (N = 61) [8,9,10,11,12,13,14,15,16,17,18,19,20,21,22,23,24,25,26,27,28,29,30,31,32,33,34,35,36,37,38,39,40,41,42,43,44,45,46,47,48,49,50,51,52,53,54,55,56,57,58,59,60,61,62,63,64,65,66,67,68], PBM (N = 15) [69,70,71,72,73,74,75,76,77,78,79,80,81,82,83], stem cells (N = 17) [84,85,86,87,88,89,90,91,92,93,94,95,96,97,98,99,100], and extracellular vesicles including exosomes (N = 8) [101,102,103,104,105,106,107,108].

### 3.1. Platelet-Rich Plasma (PRP)

PRP—an autologous administration of a plasma preparation enriched in platelets above whole blood levels—is the most common application of regenerative medicine in hair restoration. In 2019, the ISHRS (International Society of Hair Restoration Surgery) published its position supporting the use of PRP, evidenced by the significant hair density improvements [1]. However, further works are needed to establish the proper use of PRP due to unstandardized quality control measures, preparation and administration protocols, subjective outcome assessments, and lack of large randomized controlled trials [1,2,109,110,111].

Behind these uncertainties are intrinsic and extrinsic variables. On a patient-by-patient basis, the amount of growth factors released by platelets is unpredictable, irrespective of the PRP collection system used [112]. The active component of PRP is more than just growth factors released by platelets, and its exact composition can vary by patient characteristics such as age and comorbidity [2]. PRP collection systems are not equal in their whole blood volume requirements, number of centrifugation cycles, addition of anticoagulants and activation, and platelet yield [2]. An investigation by Inyang et al. found substantial variabilities among three PRP collection systems, of which two systems produced PRP with lower platelet concentrations than whole blood (i.e., platelet-poor plasma [PPP]), while one system co-concentrated white blood cells [113]. PRP can be processed and formulated into leukocyte-poor PRP (i.e., pure PRP), leukocyte-rich PRP (L-PRP), platelet-rich fibrin (PRF; free of anti-coagulants), or leukocyte-rich PRF [2].

#### 3.1.1. Mechanisms of Action

Platelet release of paracrine signaling molecules—such as growth factors, extracellular vesicles (exosomes), inflammatory factors, membrane permeability factors—through α- and δ-granules is thought to be the main mechanism of action of PRP [2,114]. This process can often be facilitated by chemical or mechanical activation methods. In particular, platelet-derived growth factor—subunit B (PDGF-BB), epithelial growth factor (EGF), and vascular endothelial growth factor (VEGF) are thought to promote hair growth [2,111].

In a histopathologic examination of AGA patients treated with PRP, an increased number of hair follicle progenitor cells, enhanced cell proliferation, as well as elevated β-catenin protein levels, were observed [35]. Furthermore, a positive correlation was observed between increases in hair density and PRP GDNF (glial cell-derived neurotrophic factor) levels [46,115], and between increases in hair diameter and PRP PDGF-BB levels [115]. Terminal hair density was also positively correlated with platelet counts [33].

Recent advances have highlighted the positive role of leukocytes in mediating the release of growth factors, leading to increased support for leukocyte-rich PRP [2,111]. Due to the potential interference of wound healing caused by anticoagulants, the use of PRF is also gaining recognition, which simulates a fibrin matrix with improved release kinetics for growth factors [2]. An injectable PRF formulation has been developed to overcome its gel-like consistency [116]. In contrast, erythrocytes—associated with bruising and tissue damage by releasing reactive oxygen species—should be minimized in PRP preparations [2].

#### 3.1.2. Clinical Studies

Due to the ongoing issue of study quality concerning PRP investigations [114,117], this section will focus on studies evaluated in reference to the reporting recommendations outlined by Kon et al. [118], Sharun et al. [119], and Harrison et al. [120], alongside objective outcome assessments for hair density and/or hair thickness and Level of Evidence (see Table S1) [121]. Authors deemed a study to be of poor quality when platelet concentrations, obtained before and after PRP preparation, were not reported [122]. The ideal PRP platelet concentration may be between 1 and 1.5 million per µL [123].

A summary of clinical efficacy is shown in Table 1, totaling 388 AGA patients. PRP has generally shown effectiveness as a treatment for AGA; however, there remains a paucity of large, high-quality studies to better guide clinical use [2,114,117]. Improvement in the total hair density was observed in six studies [27,35,37,47,53,67], which was more pronounced when the outcome assessment was conducted at 6 months after treatment (+23.1 to +49.4 hairs/cm^2^) instead of 3 months (+8.1 to +19 hairs/cm^2^). This reflects a need for longer follow-up periods in future studies.

There are conflicting findings regarding the utility of PRP activation. While VEGF concentration was shown to be unaffected by calcium activation [15], another study reported a higher degree of total hair density improvement following calcium activation [47]. The addition of an exogenous activator may compromise the autologous definition of PRP treatment and accelerate the release of growth factors in short bursts compared to endogenous activation [114,123].

Among new PRP formulations, L-PRP has been tried in two studies [30,64]. After three monthly injections of L-PRP, Batni et al. reported improvements in physician scoring and negative hair pull tests for all patients [64]. In a case report, after receiving one L-PRP injection followed by a booster, an increase in vertex hair coverage and hair thickness was observed [30].

PRF has been tried in four studies—demonstrating improvements in hair density for up to 6 months—albeit without reporting the platelet counts [44,45,55,62]. Potential issues in PRF treatments include the approximate 20 min time window for injection to prevent clotting, and a lower platelet concentration compared to conventional PRP [44,123].

Pure or leukocyte-poor PRP has also demonstrated comparable efficacies [30,37,53]. Peng et al. utilized the whole blood separator as part of the apheresis procedure to prepare pure PRP for administration to 15 female AGA patients [37]. After 3 monthly injections, an increase in hair density by 19 hairs/cm^2^ was observed [37].

Frequencies of PRP treatment are most commonly reported as three sessions at monthly intervals. Hausauer et al. introduced the protocol of three sessions per month followed by a booster at month 6, which was shown to be more effective than two sessions every three months [124]. Recently, the concept of a personalized treatment approach entailing an initial loading dose (“induction”), repeated for patients not achieving an optimal response, followed by maintenance therapy has been proposed [2,114]. Paththinige et al. reported the administration of three monthly PRP treatments followed by a booster treatment after 2 months, demonstrating a gradual increase in the total hair density up to month 6 (102.2 to 161.8 hairs/cm^2^) [36].

#### 3.1.3. Adverse Events

In contrast to other biologics, one advantage of PRP is its favorable safety profile attributed to its reduced immunogenicity [2]. Common adverse events include application site reactions such as erythema, swelling, scalp sensitivity, pruritus, pain, and bruising [14,27,33,37,43,48,53,67]. In a pilot study, Linkov et al. evaluated the use of a needleless, jet propulsion device for transdermal PRP treatment in 14 AGA patients [25]. After three monthly sessions, each delivering 5 mL of PRP, none of the patients reported pain, and most reported being comfortable during and after the procedure [25].

#### 3.1.4. Controversies

Both PRP and its collection systems are subject to regulatory oversight, which differs significantly per country [125]. In the U.S., the Center for Biologics Evaluation and Research under the Food and Drug Administration (FDA) oversees PRP as a biologic and a blood product [125,126]. Since it is not classified as human cells, tissues, or cellular or tissue-based products (HCT/Ps), and is considered minimally manipulated, the autologous use of PRP is generally exempt from Biologics License Applications, which would entail providing data from animal studies and clinical trials [125,126]. This leaves the current regulatory pathway for PRP being the 510(k) clearance—requiring the PRP collection device to be safe and “substantially equivalent” to existing devices—with less emphasis on its clinical efficacy [126]. Currently, PRP can be offered by not only hair transplant surgeons and dermatologists, but also general practitioners and non-physicians [127]. In aesthetic dermatology, patients seen by non-physicians—concerning those who operate outside of a clinic—are more likely to experience adverse events [128].

### 3.2. Photobiomodulation (PBM)

Low-level laser (light) therapy, or PBM, entails irradiating the scalp with red (600–700 nm) or near-infrared lights (780–1100 nm) to stimulate hair follicle growth without heating [3,129]. In 2007, a handheld laser device was cleared by the US FDA as a treatment for male AGA patients with Hamilton–Norwood grades of IIa-V and Fitzpatrick skin types of I-IV [130]. Since then, numerous similar devices have been made available with both LED (light-emitting diodes; non-coherent) and laser (coherent) as the light source, allowing AGA patients to access a simple, non-invasive procedure that can be administered at home [129,131].

Due to heterogeneities in the treatment protocol and device designs (e.g., helmets, headbands, combs), as well as scarcity of long-term studies, a consensus is yet to be reached on whether PBM can be effective as a monotherapy for AGA [3,116,132]. A recent Delphi consensus exercise found PBM to be a safe treatment in adults and can be effective in inducing hair regrowth; however, authors also emphasized the importance of consistently reporting key device settings (e.g., fluence, wavelength) to ensure transparency and reproducibility across studies [3].

#### 3.2.1. Mechanisms of Action

Through the absorption of red or near-infrared lights, an activated cytochrome c oxidase is a key driver that enhances mitochondrial adenosine triphosphate (ATP) production, and is thought to be the main effector in hair regrowth [3,133]. An increase in cyclic adenosine monophosphate (AMP) is also observed, which may downregulate inflammation [133], corroborated by histopathologic examination [69]. Experimentally, PBM was able to counteract the inhibitory effects of dihydrotestosterone (DHT), a potent androgen central to AGA pathogenesis, in dermal papilla cells [134]. This effect was more pronounced at lower DHT concentrations and was dose-dependent, with an optimal energy density of 8 J/cm^2^.

Due to changes in the mitochondrial structure, an influx of reactive oxygen species, Ca^2+^ and nitric oxide (NO) may regulate intracellular signaling pathways; NO is also considered a vasodilator and a DHT inhibitor that may facilitate hair regrowth [133,135]. The choice of wavelength can determine the degree of cytochrome c oxidase activation and thus affect the efficacy of PBM [133]; for AGA, lower wavelengths are generally preferred, but a consensus has not been reached.

#### 3.2.2. Clinical Studies

PBM studies in AGA patients need to be examined with closer scrutiny, as close to 70% of patients may not experience clinical improvements to a similar extent as oral finasteride, and results may vary significantly between studies [4]. Thus, quality of the included studies was evaluated based on the Level of Evidence [121], and reporting of key device parameters in reference to previous recommendations by Maghfour et al. [3], and the US FDA draft guidance on 510(k) submissions [136] (Table S2).

Summary of included studies totaling 970 patients is shown in Table 2. Conflicting findings were observed in sham device-controlled trials, indicating potential confounding variables such as device settings and patient characteristics [70,79]. In a 16-week study, a once every 2 days application of PBM—emitting both laser and LED with a wavelength of 655 nm—led to significant improvements in hair density (41.9/cm^2^) and thickness (7.5 µm), while patients receiving the sham device treatment had a minimal increase in hair density (0.7/cm^2^) with a decrease in hair thickness (−15 µm) [70]. In contrast, Thomas et al. randomized patients to be treated daily using three different PBM devices (red light [1.6 mW/cm^2^], blue light [1.6 mW/cm^2^], or red and blue light [3.3 mW/cm^2^]) or a sham device [79]. Results obtained at week 26 showed no significant improvements in hair density, which the authors attributed to the large number of protocol violations [79].

When reporting device parameters, the fluence (i.e., dose) is recommended as it indicates the total amount of energy delivered per unit area; however, this was only provided in 3 studies with a considerable degree of variation, ranging from 40–120 J/cm^2^; similarly, reported power densities also largely varied from 1.3–90 mW/cm^2^. As per the biphasic dose response to PBM, a smaller dose may reduce tissue penetration, rendering the treatment ineffective, while a larger dose may cause inhibitory, thermal effects [4]. Another potential confounding variable is the distance from the light source to the treated area, affecting power density [3], which was reported in only one study [83].

Besides device parameters, patient characteristics such as skin and hair color affecting light penetration have not been accounted for. As melanin—a chromophore that absorbs light—is found in both skin and hair, which hinders light absorption into tissues, it has been suggested that the ideal patient population for PBM is those with fair skin and blonde/bleached hair [4].

In a real-world study of 597 AGA patients, PBM (650 nm) was administered once every 2 days for 38–40 weeks [81]. Efficacy results—based on a 6-point scoring system that includes reduction in (1) sebum, (2) dandruff, (3) erythema or (4) daily hair loss and increase in (5) hair density, or (6) hair thickness—showed that PBM was “significantly effective” in only 23.3% (132/597) of patients (scores 4–6) [81]. This finding is in agreement with the analysis by Keene (2015) that about 70% of AGA patients do not adequately respond to PBM treatments [4]. Physicians reviewing PBM studies should remain cognizant that the results may not be generalizable due to differences in light source (LED/laser), wavelength, fluence, power density, distance from the light source to the scalp, as well as patient characteristics such as hair color and Fitzpatrick skin type.

#### 3.2.3. Adverse Events

No serious adverse events were reported. Self-limited application site reactions include scalp tenderness, heat sensation, erythema, pruritus, hyperpigmentation, and headache (for helmet-type devices) [77,78,79,83]. In the absence of safety data on ocular damage, the use of protective goggles or a device with skin contact sensors may be recommended [136,137].

#### 3.2.4. Controversies

Since 2007, the wide accessibility of PBM devices has made self-administered home therapies easier for patients. However, most of these marketed devices are not covered by insurance, have varying degrees of clinical efficacy, and sometimes lack a clear indication or protocol that can mislead consumers [4,131]. Furthermore, none of the devices have been FDA cleared for those with Fitzpatrick skin types V and VI [137]. Physicians are recommended to prescribe PBM devices only when they have received US FDA 510(k) clearance with empirical evidence on efficacy [4]. When the device parameters are not correctly adjusted—especially concerning patients who purchased these devices online without a prescription—there is a risk of damaging and scarring hair follicles [138].

### 3.3. Stem Cells

Mesenchymal stem cells are characterized by their ability to self-renew and regenerate. In practice, stem cell treatments can be administered by ways of transplantation, where whole cells derived from tissues are used, or stem-cell conditioned media (CM), where bioactive molecules released by stem cells (i.e., secretome), such as cytokines and growth factors, are harvested, or stem cell-derived extracellular vesicles or exosomes (see Section 3.4.) [116].

Despite the wide range of tissue sources for stem cells, the adipose tissue is preferred for dermatological applications due to the ease of collection, non-invasive procedure, safety due to its autologous use, and high cell yield [116]. Other sources of stem cells being investigated include the umbilical cord blood; however, it cannot be administered as an autologous treatment and has potential ethical considerations [139]. The hair follicle, dermal papilla, and bone marrow can also be used to extract stem cells [140].

Stem cell therapies are under the jurisdiction of the FDA’s Center of Biologics Evaluation and Research [125]. FDA pre-market approval may not be required for certain products considered minimally manipulated and intended for homologous use [125]. Otherwise, these products will need approval through the Biologics License Application [125]. For AGA, it remains unclear whether non-hair follicle stem cells would be considered homologous despite growing evidence that the scalp adipose plays a role in follicular cycling. Currently, none of the stem cell products are approved for dermatological indications.

#### 3.3.1. Mechanisms of Action

Although stem cells were originally thought to have a direct-acting mechanism of action through targeting damaged sites and replacing defective cells by differentiation, recent works have acknowledged the role of paracrine signaling factors and immunomodulatory functions [141,142]. Still, the exact mechanisms of action remain unclear.

Adipose tissues may play a role in hair cycle regulation. In particular, the scalp dermal white adipose tissue is closely linked to the hair growth cycle through signaling interactions [143]. During the anagen phase, the maturation/enlargement of adipocytes causes the expansion of the dermal white adipose tissue, while during the catagen phase, matured adipocytes undergo cell lysis or turnover into progenitor cells, causing the thinning of the dermal white adipose tissue [143]. Matured adipocytes express BMPs (bone morphogenic proteins), which may function in maintaining the hair inductivity in dermal papilla cells, as well as upregulate angiogenesis through modulating the expression of the VEGF receptor [143]. Progenitor adipocytes help maintain PDGFA (platelet-derived growth factor subunit A) expression in dermal papilla cells, which plays a role in the activation of hair follicle stem cells [143].

CM from stem cells also contains growth factors such as BMPs, VEGF and PDGF [140]; the release of these growth factors may be induced by exposing stem cells to low-oxygen (hypoxia) conditions or adding vitamin D3 [140].

#### 3.3.2. Clinical Studies

Clinical translation of stem cells is an ongoing effort with many technical nuances, especially concerning products that were manipulated or expanded in a laboratory setting. In studies where stem cells are obtained through punch biopsies, the low cell count often necessitates an in vitro expansion step where cells are incubated and passaged [87,97]. This could lead to unforeseen morphological and genetic changes affecting their differentiation ability [144]. Due to methodological and sample source variations in isolating mesenchymal stem cells, the International Society for Cell Therapy has recommended cellular and molecular characterizations as a reporting standard [145]. However, these are often not feasible in clinical studies, highlighting the need to establish standards for stem cell manufacturing and quality control.

Summary of efficacies reported in stem cell studies is shown in Table 3; studies employing subjective outcome assessments were excluded. Overall, results appear to significantly vary between studies, with changes in total hair density at month 12 ranging from a decrease by 2.1/cm^2^ to an increase by 30/cm^2^ [87,88]. Sources of stem cells included scalp biopsies and lipoaspirate for autologous preparations [97,99], while one study reported a topical allogenic preparation containing lysates of adipose-derived stem cells (ADSCs) [84].

Hair follicle-derived stem cells (HFSCs) have been tried successfully as an autologous treatment in controlled studies, although the durability of response with single injections appears to be less than one year [97,98]. Tsuboi et al. examined the utility of dermal sheath cup (DSC) cells obtained from skin biopsies of the occipital region in AGA patients [97]. After in vitro expansion, DSC cells were administered via a single injection into the vertex area [97]. Four vertex regions of each patient were randomized to receive three different doses of DSC cells or placebo; at months 6 and 9, significant increases in hair density were observed using the lower dose (3 × 10^5^ DSC cells), which dissipated by month 12 [97]. In a follow-up transcriptomic analysis, DSC cells from treatment responders showed an upregulation of genes implicated in cell adhesion and migration, which reflects a better ability to reach miniaturized hair follicles, thereby enabling cell differentiation into dermal papilla cells [96]. However, in another study using the same dose (3 × 10^5^ DSC cells), where patients were treated twice over 3 months, no improvement in hair density was observed at month 12 [87].

In a controlled, split-scalp study, Gan et al. harvested hair follicles from the occipital area of AGA patients, which were used for stem cell extraction without in vitro expansion [98]. Two vertex regions received injections of either approximately one million HFSCs or saline [98]. Compared to saline, HFSC injections led to a significant increase in hair diameter by month 3, which was more pronounced for thinner hair follicles with diameters less than 60 µm; however, this effect appeared transient as hair diameters returned to baseline measurements by months 6 and 9, which authors attributed to the waning survival and differentiation ability of HFSCs outside of their original niche [98].

Four studies utilized a device that mechanically homogenizes punch biopsy specimens obtained from AGA patients, with the resultant cellular suspension (without in vitro expansion) expressing markers characteristic of HFSCs [86,88,89,90]. In two studies where a single injection was administered, an improvement in hair density was observed up to month 12 (increase by 30/cm^2^ for males and 28/cm^2^ for females) [88,89]. When administered in multiple sessions, one study reported an improvement of hair density by 23.3/cm^2^ at week 58 [86]. Despite these promising results, a recent study utilizing the same device reported no significant changes in hair density or diameter [90].

More consistent findings were observed in studies reporting treatments based on lipoaspirates [84,85,99], which typically yield higher cell numbers than biopsies. In 30 AGA patients, intradermal injections of autologous total-stromal cells resulted in a 16% improvement in hair density and a 51.3% improvement in hair diameter at month 6 [99]. When emulsified adipocytes were further purified to concentrate ADSCs by eliminating background materials (e.g., oil, water, fibrous matter), a study of 10 AGA patients reported a 22.4/cm^2^ increase in mean hair density at month 12, after three monthly injections [92]. In a randomized, controlled study, Tak et al. developed a topical allogenic product based on the adipose tissue obtained from healthy donors [84]. After twice-daily applications for 16 weeks, a significant improvement in hair density was observed compared to control (3.8/cm^2^ vs. 1.4/cm^2^) [84].

In sum, direct-acting stem cell treatments using HFSCs have shown inconsistent clinical efficacy, warranting further research. The lack of a durable treatment response may be attributed to low cell counts from biopsies, low injection frequency, or loss of cell viability over time. In comparison, although ADSCs have demonstrated long-term benefits, possibly through paracrine mechanisms, the number of published studies remains limited. The development of an allogenic product may help reduce study variability.

#### 3.3.3. Adverse Events

Injection site reactions were commonly reported as pain, swelling, folliculitis, erythema, erythrosis, purpura, and bleeding, which were self-limited [87,97,98,99]. Tumorigenesis in case of stem cell transplants (especially induced pluripotent stem cells), and infection and graft-versus-host disease in case of allogeneic stem cells, are important safety concerns that have not been fully addressed [141]. Alternatively, the use of stem cell CM or extracellular vesicles may help to reduce these risks, but further research is needed [141].

#### 3.3.4. Controversies

Early advocates for stem cell therapies often assumed its safety attributed to its autologous or allogenic sources, and justified its widespread adoption—without regulatory approval—based on the unsubstantiated idea that stem cells have the universal ability to sense their surroundings and perform repairs through differentiation [146]. The lack of regulatory approval has inadvertently led to a concerning trend of unproven stem cell therapies being administered worldwide, which could impede future research efforts and create additional regulatory hurdles [147]. The FDA has outlined key priority areas in developing the regulatory science behind stem cells and other regenerative therapies, such as standardizing manufacturing and quality control measures, timely evaluation of new products, active collection of clinical data, and raising public awareness [148]. Following the end of a grace period in 2020—when stem cell manufacturers could voluntarily engage with the FDA to ensure compliance—the agency now enforces its regulation on all stem cell therapies [148].

### 3.4. Exosomes

Extracellular vesicles (30–1000 nm in diameter), secreted by a variety of cell types, including stem cells, keratinocytes, fibroblasts, outer root sheath cells, and dermal papilla cells, can function as cargo-delivering vehicles between cells and regulate intracellular signaling [140]. Since paracrine signaling plays an important role in the therapeutic potential of cell-based therapies, including stem cells, it is believed that extracellular vesicles represent a key functional component that may serve as a “cell-free” alternative treatment [140].

**Figure 3:**
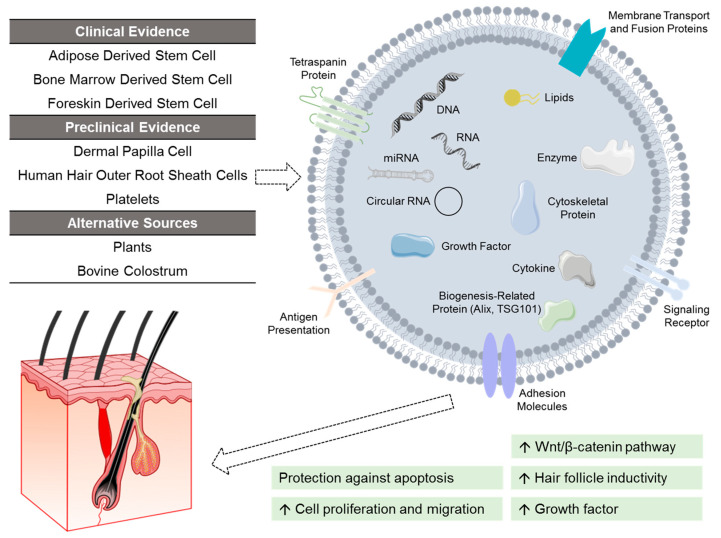
Investigational exosomes treatment for androgenetic alopecia. Exosomes are characterized by surface transmembrane proteins, and function by delivering cargos to recipient cells. These cargos (e.g., nucleic acids, lipids, growth factors) vary depending on the source of exosomes. At present, exosomes derived from adipose stem cells, bone marrow stem cells, and foreskin stem cells have shown clinical efficacy. Other investigated sources of exosomes include dermal papilla cells, human hair outer root sheath cells, platelets, as well as alternative non-human sources like plants and bovine colostrum.

In particular, exosomes (30–150 nm in diameter) have been studied for their application in hair restoration (Figure 3) [5]. Despite pre-clinical studies demonstrating the promise of exosomes [5], the science is still unclear, and a regulatory pathway has yet to be developed due to their inherent complexity and heterogeneity [149]. The U.S. FDA currently regulates exosomes as a biologic and has not approved any product to date [150].

#### 3.4.1. Mechanisms of Action

Through the delivery of bioactive substances to target cells (e.g., RNA, signaling lipids, growth factors) protected by its lipid bilayer, exosomes serve as a mediator of cell communication and can regulate the hair growth cycle [5]. Exosomes derived from dermal papilla cells—containing microRNAs that regulate gene expression—have been shown to upregulate the Wnt/β-catenin pathway and increase the proliferation of hair follicle stem cells [5]. Exosomes isolated from ADSCs also demonstrated the ability to activate the Wnt/β-catenin pathway and promote hair follicle induction in dermal papilla cells [5]. In dermal papilla cells challenged by DHT, ADSC-derived exosomes were shown to alleviate DHT-induced growth inhibition effect by delivering microRNAs that inhibit the TGF-β1/SMAD pathway [151]. This effect translated into increased hair follicle growth both ex vivo and in a mouse model.

Other promising sources of exosomes demonstrating similar pre-clinical efficacy include keratinocytes, outer root sheath cells, and amniotic fluid stem cells [5]. Recent research has also highlighted the potential of xenogeneic exosomes, including those derived from milk (e.g., bovine colostrum) and from plants (e.g., rose stem cell, ashwagandha seeds) [5,152].

In patients who received three monthly topical applications of rose stem cell-derived exosomes, ultrasound examination was conducted to compare the treated area with surrounding and contralateral tissues [107]. In the exosome-treated region, hair follicles appeared more prominent with visible new hair shafts, and an increase in dermal vascularity was observed.

#### 3.4.2. Clinical Studies

Significant heterogeneities exist in the reporting of exosome treatment. In addition to naïve exosomes, exosomes can be bioengineered to alter their functional contents and targeting [153]. Processing conditions (e.g., isolation technique, thawing procedure) and quality control measures (e.g., surface marker detection, viral contamination) also affect the integrity of exosomes, which are currently unstandardized. Although recent pilot studies have evaluated the effects of plant-derived exosomes in AGA patients [154,155], interpreting these results is challenging due to exosomes being admixed with other active ingredients such as growth factors and peptides [156].

Exosomes isolated from ADSCs were tried in four studies [102,103,104,106]. In a placebo-controlled study, AGA patients were randomized to receive microneedle-assisted administrations of either ADSC-exosomes or saline for 3 sessions spaced 4 weeks apart [103]. At week 12, exosome-treated patients showed greater improvements in mean hair density (35/cm^2^ vs. 3/cm^2^) and thickness (13.0 µm vs. 1.8 µm) [103]. Similar improvements, up to week 24, were also reported in single-arm studies following 10–12 administrations of topical ADSC-exosomes [102,104]. By contrast, a single administration was also reported to significantly improve hair density by week 48 (35/cm^2^ [±6.5]), further supporting its therapeutic potential [106].

An ongoing U.S.-based trial is investigating an umbilical cord-derived exosome product to be administered with microneedling for AGA (NCT06482541). Experimentally, hair follicles of DHT-challenged mice showed transition into the anagen phase, following administration of exosomes from human umbilical cord mesenchymal stem cells [157]. This effect was possibly mediated by improving intercellular communications between hair follicle cells and the dermal papilla [158]. A topical hydrogel-based preparation was also developed, demonstrating similar effects in a DHT-challenged mouse model [159].

Despite safety concerns, intradermal injections of exosomes derived from mesenchymal stem cells were tried in two studies [101,105]. One study administered meso-injections of exosomes isolated from foreskin-derived mesenchymal stem cells [101]. After one session, hair density increased from 149.7/cm^2^ to 157/cm^2^ at week 12.

A recent study by Cao et al. hypothesized that erythrocytes—the largest cell population undergoing continuous self-renewal—can integrate into the skin and hair, delivering keratins and hemoglobin through their extracellular vesicles [108]. In a preliminary investigation involving 9 AGA patients, autologous erythrocyte-derived extracellular vesicles were injected subcutaneously. After three monthly sessions, significant improvements in hair density were reported at month 6 [108].

#### 3.4.3. Adverse Events

No serious adverse events were reported in the included studies [101,102,103,104,105]. Microneedle-assisted delivery was associated with swelling and erythema, which occurred at a similar frequency between the treatment group and the placebo group [103]. Pricking and skin tingling were also reported [102,104]. In patients who received intradermal injections, self-resolving pain and swelling were reported [105].

Due to the current lack of understanding of exosomes, concerns about serious adverse events such as malignancies, infections, and immune reactions have been raised [149]. Substandard manufacturing conditions of a placenta-derived exosome product have been linked to cases of infection, including sepsis [150,160].

#### 3.4.4. Controversies

Despite a weak evidence base, an unclear safety profile, lack of regulatory approvals, and public health warnings, commercialized exosome or extracellular vesicle products can be found in countries such as the United States, Canada, the European Union, and Japan [149,161,162]. Rahman et al. evaluated the commercial claims of eighteen exosome manufacturers, including human-, animal- and plant-based exosomes [156]. Their findings showed a common lack of disclosures, including clinical data, quality control measures, active ingredients, and product source [156]. Among these products, the composition and concentrations of active ingredients such as growth factors and miRNAs also varied [156]. Despite an overall positive consumer sentiment globally, driven in part due to online influences from both medical professionals and laypersons, there is a growing need to strengthen the scientific evidence of exosomes, all the while ensuring transparency and alignment with health regulations [156]. Physicians should exercise caution when reviewing exosomes and related cell-based products until more research is done.

## 4. Conclusions

Regenerative medicine has demonstrated promising therapeutic potential for inducing hair regrowth. However, there are conflicting findings in the current literature, which is due in part to the lack of standardization and transparency in reporting product composition and treatment protocols. In real-world practice, these modalities are often tried in combination—such as with microneedling, topical therapies (minoxidil 5%), oral antiandrogen medications (finasteride, dutasteride, spironolactone), or hair transplantation—resulting in potential synergistic effects that are not captured in monotherapy trials and are beyond the scope of this review. Without adequate data, it would be difficult to draw any definitive conclusions on the utility of regenerative therapies for AGA, and patient safety must remain paramount when considering their use.

## Figures and Tables

**Table 1:** Summary of clinical efficacy from selected PRP studies.

Study	No. Patients	AGA Severity	Formulation (Activation)	Change in Total Hair Density (n/cm^2^)	Other Clinical Outcomes
Singh [47]	80	HN: II–VI	- (Ca gluc)	Month 6: 27.7 (11.3) *	Increase in hair thickness
	80	HN: II–VI	- (None)	Month 6: 23.1 (9.6) *	Increase in hair thickness
Sorce [48]	54	HN: III–VI Ludwig: I–III	- (None)	NR	Increase in hair thickness
Batni [64]	46	Mild (N = 8) Moderate (N = 26) Severe (N = 12)	L-PRP (None)	NR	Negative hair pull test
Moftah [33]	40	Ludwig: I–II	- (None)	NR	Increase in terminal hair density and terminal to vellus hair ratio
Balasundaram [53]	32	HN: III–IV	Pure PRP (None)	Week 12: 8.1 * (*p* = 0.001)	Increase in terminal hair density
Pakhomova [35]	23	HN: II–IV	- (CaCl_2_)	Month 7: 44.6 (*p* < 0.001)	Increase in hair thickness and telogen hair
Budania [68]	22	HN: 3.2 ± 0.6	- (Ca gluc) (Low temp)	NR	Increase in terminal to vellus hair ratio
Bruce [67]	20	Ludwig: I–II	- (None)	Week 12: 10 (−11, 22) **	Increase in vellus hair density
Okita [34]	20	NR	- (Ca gluc)	Month 3: No improvement	No improvement in terminal hair density and terminal to vellus hair ratio
Singh [27]	19	HN: II–V	- (Ca gluc)	Month 5: 49.4 (5.8) * (*p* < 0.001)	-
Peng [37]	15	Ludwig: >II	Pure PRP (None)	Month 3: 19	-
Wei [54]	15	BASP: Mild/Moderate to Moderate/Severe	- (None)	Month 3: No improvement	No improvement in hair thickness
Maletic [30]	1	HN: IV	Pure PRP (Calcium)	NR	Increase in hair thickness Increase in 2-hair and 3-hair FU
	1	HN: III (vertex)	L-PRP (Calcium)	NR	Increase in hair thickness Increase in 3-hair FU

Note: Studies reporting combinational therapies, or without the reporting of platelet enrichment, were excluded. BASP, basic to specific classification; CaCl_2_, calcium chloride; Ca gluc, calcium gluconate; FU, follicular unit; HN, Hamilton–Norwood; L-PRP, leukocyte-rich PRP; NR, not reported. * Mean (SD). ** Median (Range).

**Table 2:** Summary of clinical efficacy from selected PBM studies.

Study	No. Patients	AGA Severity	Device Type Wavelength (nm)	Power Density (mW/cm^2^) Fluence (J/cm^2^)	Change in Total Hair Density (n/cm^2^)
Qiu [81]	597	BASP: mild to severe	Helmet 650	NR NR	Week 38–40: 23.3% significantly effective *
Kim [72]	49	HN: I–VI Ludwig: I–III	Helmet 630–970 **	1.3 NR	Week 24: 15
Thomas [79]	39	HN: II–V Ludwig: I–II	Cap 625–660	1.6 NR	Week 26: −2.9 ± 46.5 (*p* = NS)
	41	HN: II–V Ludwig: I–II	Cap 425	1.6 NR	Week 26: 13 ± 55.6 (*p* = NS)
	38	HN: II–V Ludwig: I–II	Cap 425/625–660	3.3 NR	Week 26: 10.7 ± 67 (*p* = NS)
Neema [83]	37	HN: II–VI	Helmet 633 ± 10	80 ± 10% NR	Month 6: 104.8 ± 22.7 to 130 ± 25.7 (*p* < 0.001)
Liu [71]	30	Ludwig: I–III	Helmet 650	NR NR	Month 6: 79.3 ± 5.4 to 104.8 ± 5.4 (*p* < 0.001)
Yoon [70]	30	HN: II–V Ludwig: I–II	Helmet 655 ± 5	2.5 NR	Week 16: 41.9
Lodi [82]	20	HN: III–VI	NR 417 ± 10	60 ± 20% 120	Week 16: 106 ± 66 to 117 ± 69 (*p* = 0.001)
Chandrashekar [74]	20	HN: I–V Ludwig: I–III	Handheld Applicator 675	NR NR	Month 4: 115.8 ± 26.2 to 135.7 ± 33.4 (17.1%)
Sorbellini [80]	17	HN: I–III Ludwig: I–II	Handheld Applicator 675	NR NR	Month 5: 249 ± 68 to 281 ± 68 (13.2%)
Tantiyavarong [78]	17	HN: III–IV Ludwig: I–II	Helmet 633	50 40	Month 6: 8.2 (1.9, 14.4) ^§^
			Helmet 522	50 40	Month 6: 7.7 (1.0, 14.4) ^§^
Eitta [76]	15	HN: II–IV	Helmet 655/650–670	NR NR	Month 6: 109.5 ± 15.8 to 108.5 ± 11.5 (*p* = NS)
Amer [77]	13	NR	Helmet 655	NR NR	Week 29: 222.3 ± 33.5 to 252.8 ± 30.4 (*p* < 0.05)
Cao [75]	7	HN: III–V	Comb 630 ± 5	90 60	Week 24: −12 ± 16.2

BASP, basic and specific; HN, Hamilton–Norwood; NR, not reported; NS, not significant. * Patients were scored based on 6 domains: reduction in sebum, dandruff, erythema, or daily hair loss, and increase in hair density or hair thickness. ** Device emitted three different wavelengths: 630–690 nm, 820–880 nm, 910–970 nm. ^§^ Change in non-vellus hair density reported as mean difference (95% CI).

**Table 3:** Summary of clinical efficacy from selected stem cell studies.

Study	No. Patients	AGA Severity	Intervention	Regimen	Change in Total Hair Density (n/cm^2^)
Tsuboi [97]	62	HN: III–VI S: III–VI	DSC cells (Autologous)	Single 1 mL injection (3 × 10^5^—7.5 × 10^6^ cells) per 2 cm^2^	Month 9: 3.6 (CI: 1.6, 5.7) (*p* < 0.05) *
Gentile [88]	60	HN: I–III Ludwig: I–II	HFSC (Autologous)	Single interfollicular infusion (0.1 mL/cm^2^)	Month 12: 30 ± 5 (male); 28 ± 4 (female)
Gan [98]	50	HN: II–V Ludwig: I–III	HFSC (Autologous)	Multiple 100 µL injections (1 × 10^5^ cells) per 1 cm^2^	Month 3: increase in hair diameter **
Harada [87]	36	HN: III–VI S: III–VI	DSC cells (Autologous)	Two sessions of multiple intradermal injections (total volume: 12 mL [3.6 × 10^6^ cells]) per 105 cm^2^ at Months 0 and 3	Month 12: −2.1 ± 3.5
El-Khalawany [99]	30	HN: I–VI Ludwig: I–III	Total-stromal cells (Autologous)	Single intradermal injection 0.1 mL/cm^2^	Month 6: 130.8 ± 14 to 151.9 ± 22.3 (*p* < 0.001)
Gentile [86]	27	HN: II–V Ludwig: I–II	HFSC (Autologous)	Three sessions of interfollicular infusion (0.2 mL/cm^2^) every 45 days	Week 58: 23.3
Legiawati [91]	20	HN: III–VI	ADSC-CM (Allogenic)	Three monthly sessions	Week 12: 40.19 ± 30.07
Tak [84]	18	BASP: M2, C2, U1, V1, F1	ADSC-CE 1% (Allogenic)	Topical application 2 ml twice daily	Month 16: 3.8 ± 3
Vincenzi [90]	17	HN: I–III Ludwig: I–II	HFSC (Autologous)	Single injection	Month 6: increase in hair diameter
	13	HN: I–III Ludwig: I–II	HFSC (Autologous)	Two injections at Month 0 and 6	Month 12: no change
Ruiz [89]	10	NR	HFSC (Autologous)	Single injection	Month 2: 15.2 to 48.5
Zhang [92]	10	HN: III–VI	ADSC (Autologous)	Three intradermal injections (0.25 mL/cm^2^) per month	Month 12: 22.4
Nilforoushzadeh [85]	9	NR	Whole fat (Autologous)	Single injection 1 mL/cm^2^	Month 6: 100 to 230 (*p* < 0.001)

ADSC, adipose-derived stem cell; BASP, basic and specific classification; CE, constituent extract; CI, confidence interval; CM, conditioned media; DPC, dermal papilla cell; DSC, dermal sheath cup; HFSC, hair follicle stem cell; HN, Hamilton–Norwood; NR, not reported; S: Shiseido classification. * Only observed with low-dose DSC cell treatment (3 × 10^5^ cells). ** Transient increase in hair diameter at Month 3, which was not observed at Month 6 and Month 9.

## Data Availability

No new data were created or analyzed in this study. Data sharing is not applicable to this article.

## Supplementary Materials

The following supporting information can be downloaded at https://www.mdpi.com/article/10.3390/medsci14010005/s1, Table S1: characteristics and assessment of included platelet-rich plasma studies; Table S2: characteristics and assessment of included photobiomodulation studies.
